# Hybrid Compliant Musculoskeletal System for Fast Actuation in Robots

**DOI:** 10.3390/mi13101783

**Published:** 2022-10-20

**Authors:** Pieter Wiersinga, Aidan Sleavin, Bart Boom, Thijs Masmeijer, Spencer Flint, Ed Habtour

**Affiliations:** 1 Faculty of Science and Engineering, University of Groningen, Postbus 72, 9700 AB Groningen, The Netherlands; 2 Department of Aeronautics & Astronautics, The University of Washington, Seattle, WA 98195, USA; 3 The Illimited LAB, University of Washington, Guggenheim 211, Seattle, WA 98195, USA

**Keywords:** nature-inspired, robots, nonlinear, dynamics, biomimetic, antagonistic actuation, energy storage–release

## Abstract

A nature-inspired musculoskeletal system is designed and developed to examine the principle of nonlinear elastic energy storage–release for robotic applications. The musculoskeletal system architecture consists of elastically rigid segments and hyperelastic soft materials to emulate rigid–soft interactions in limbless vertebrates. The objectives are to (i) improve the energy efficiency of actuation beyond that of current pure soft actuators while (ii) producing a high range of motion similar to that of soft robots but with structural stability. This paper proposes a musculoskeletal design that takes advantage of structural segmentation to increase the system’s degrees of freedom, which enhances the range of motion. Our findings show that rigid–soft interactions provide a remarkable increase in energy storage and release and, thus, an increase in the undulation speed. The energy efficiency achieved is approximately 68% for bending the musculoskeletal system from the straight configuration, compared to 2.5–30% efficiency in purely soft actuators. The hybrid compliance of the musculoskeletal system under investigation shows promise for alleviating the need for actuators at each joint in a robot.

## 1. Introduction

It is quite remarkable to witness the massive scientific advancements in the field of biomimetic and soft robots just in the last decade [1,2,3,4,5]. Significant research has focused on engineering various compliance mechanisms to enable adequate soft actuation for nature-inspired robots [6,7,8,9,10]. Through compliance, i.e., high elastic deformation, soft actuation is achieved by distributing the loads to minimize interfacial stress concentrations [11,12]. Often, soft actuators are comprised of novel active materials and complex auxiliary circuits. Utilizing 3D printing, a variety of active materials can be leveraged, such as hydrogels [13], conductive polylactic acid [14], shape-memory alloys [15], and multiphase materials [16]. The auxiliary circuits can be thermal [16], fluidic [17], magnetic [18], or electrical [6]. Realizing soft robots into practical applications is still lagging due to performance shortcomings of their compliant mechanisms, such as low energy efficiency [19] and structural stability when bearing high loads [20,21].

**Figure 1:**
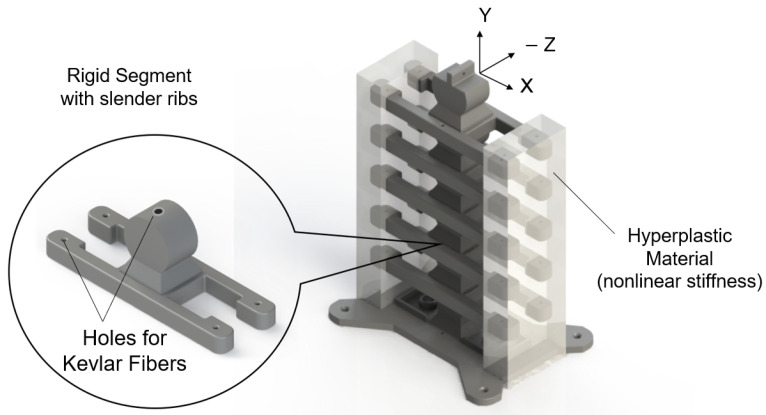
The snake-inspired musculoskeletal system consists of rigid segments (dark gray) connected with hyperelastic material (transparent sections) at the end of the ribs. Each rigid segment has a socket–disk joint and two ribs on each side. The hyperelastic material provided nonlinear elastic energy storage. The ribs transfer energy between the −X and *X* sides.

**Figure 2:**
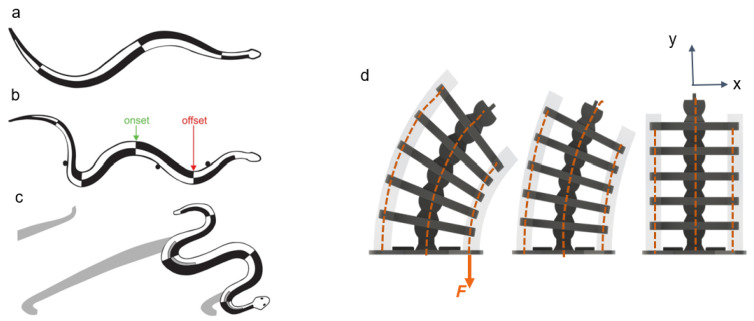
(**a**–**c**) Undulations in snakes [22]: the black-shaded areas are the muscle timing relative to bending in (**a**) swimming and (**b**) lateral undulation of a water snake and (**c**) sidewinding in a rattlesnake. The outside gray areas are past regions of static contact with the ground; note the onset and offset bending points. (**d**) Bends in our snake-inspired system: the transparent sections are the hyperelastic (soft) material connecting the ribs and providing antagonistic variable stiffness and energy storage–release. Kevlar fibers in the soft material and centerline provide actuation and prevent socket shifting, respectively.

Responding to these shortcomings, we introduce in this study a nonlinear hybrid compliant mechanism into a snake-inspired musculoskeletal architecture [22] The aim is to depart from purely soft or rigid actuation to combined rigid–soft interfaces, as shown in Figure 1 and Figure 2. The hybrid compliant mechanism allows us to maintain rotational synchronous motion [23] at the joints by taking advantage of bidirectional antagonistic stiffness actuation [23] instead of using rigid bidirectional servo motors or purely soft actuators, which are often seen in biomimetic robots [24,25,26]. Antagonistic variable stiffness actuation was achieved by placing the continuum soft (hyperelastic) materials in opposing directions to the neutral axis position for torque production about the joint (see Supplementary Materials Video S1). Hybrid compliant mechanisms are advantageous for generating high energy storage–release because they can produce a large range of motion and geometric and material nonlinearities simultaneously (Figure 2a–c) [22], as described in Section 1.1 and Section 1.2. The nonlinearities are due to the high elastic stretch in hyperelastic materials, which is key to increasing strain energy storage. The antagonistic hybrid compliant design approach accomplished two objectives: (i) improving the energy efficiency for actuating undulations over current soft actuators and (ii) producing a high range of motion while maintaining structural stability. The energy storage–release principle was demonstrated analytically and experimentally for planar undulation only (Figure 2d).

### 1.1. Nonlinear Hybrid Compliance

**Figure 3:**
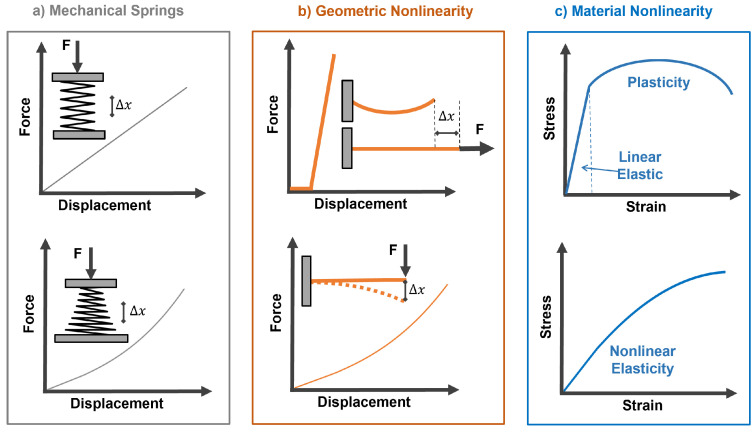
(**a**) Force–displacement response for linear (top) and nonlinear (bottom) conical springs, where F is the applied force, and Δx is the displacement; (**b**) examples of geometric nonlinearity in slack–tension (top) and bending (bottom) structural members; and (**c**) stress–strain in materials exhibiting elastoplasticity (top) and nonlinear elasticity (bottom).

Our system is designed to be nonlinear hybrid compliant, a subclass of compliant mechanisms that combines geometric and material nonlinearities [27,28,29,30,31]. Their influence on storing and releasing strain energy in biological and robotic musculoskeletal systems is underexplored [32,33,34]. Through reliance on flexible instead of rigid components, compliant mechanisms can sustain large elastic deformations to realize both high mobility and energy storage. Figure 3 provides three compliant approaches: springs, flexible structures [35], and nonlinear materials [36]. When selecting commercially available linear and nonlinear springs, designers are limited by their coiled architecture, space requirements, and force–displacement range (Figure 3a).

**Figure 4:**
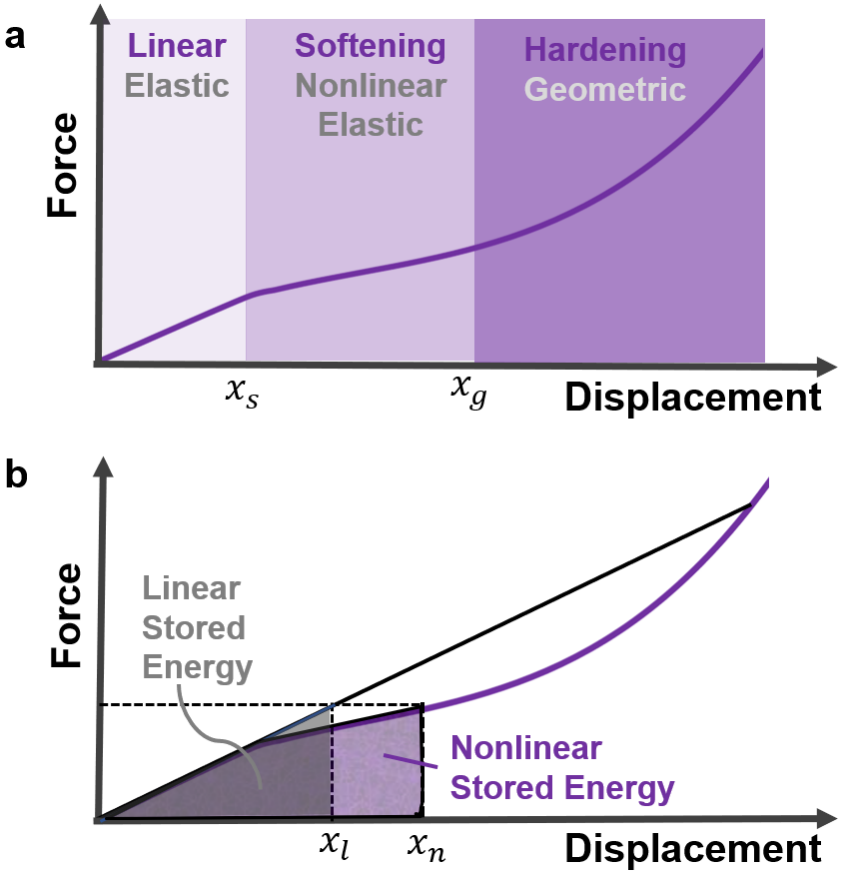
(**a**) Force–displacement curve of typical nonlinear hybrid compliance with linear softening (starts at $x_{s}$) and hardening (starts at $x_{g}$) stiffness regions; (**b**) note the linear and nonlinear displacements, $x_{l}$ and $x_{n}$, respectively, for the same force. Thus, energy storage, the area under the curve, is higher in nonlinear (purple) than in linear (gray) hybrid compliance.

The geometric nonlinearities of flexible structural elements offer an attractive alternative to conical springs. They can reach high deformation before the materials exhibit any significant departure from the linear stress–strain regime [37]. Examples of nonlinear force displacement due to slack–tension and bending of flexible structures are illustrated in Figure 3b. Common material nonlinearities ranging from elastoplasticity to nonlinear elasticity are shown in Figure 3c. Materials operating in nonlinear elastic regimes have higher strain-energy storage than linear ones, as shown in Figure 4. For a musculoskeletal design, distributed nonlinear hybrid compliance can be achieved by combining the geometric and material nonlinearities (Figure 4). In this study, the principles were demonstrated for linear and softening regimes.

### 1.2. Musculoskeletal Energy Storage–Release

Variable stiffness, which is the inverse of compliance, is directly related to its exploitation of strain energy: the area under the stress–strain or force–displacement curve. In this study, the soft continuum was hyperelastic material, where the stress–strain was nonlinear elastic (also known as dynamic softening) [38,39,40], as shown in Figure 3c and Figure 4b.

Hardening can be achieved through geometric nonlinearities and is key for increasing the speed of return (snap) from the deformed state to the neutral position upon releasing the stored bending energy. The stored elastic energy was released rapidly at ∼22 ms. The musculoskeletal system achieved ∼68% energy efficiency for executing high bending motion from a straight configuration to a 158 mm radius. Our energy efficiency exceeded that of soft actuators reported in the literature, which ranged from 2.5% to 30% [41,42,43]. The nonlinear compliance of the musculoskeletal system allowed us to eliminate the need for mechanical motors at the joints by leveraging the moments applied at the ribs.

The musculoskeletal system design and fabrication are presented in Section 2. The kinematics of the segmented system are detailed in Section 3. Section 4 describes the experimental setup and the approach for evaluating energy storage–release. The theoretical development of elastic energy storage in hyperelastic materials is provided in Section 5. The experimental and analytical results are detailed in Section 6.

## 2. Design and Fabrication

The musculoskeletal system consisted of six 3D-printed rigid segments (Figure 1) connected with hyperelastic material and Kevlar fibers. The hyperelastic material held both sides of the rigid segments of the full musculoskeletal assembly near the rib ends in the +X and −X directions (Figure 2). Additionally, Kevlar fibers ran internally through the neutral line of the structure. The rigid segments were fabricated using polylactic acid (PLA) with 35.9 MPa tensile strength and 4% maximum elongation [44]. The hyperelastic material was platinum-catalyzed silicone Ecoflex 00-30 with ∼900% rupture. The Kevlar 29 tensile strength was ∼2.75 GPa [45].

**Figure 5:**
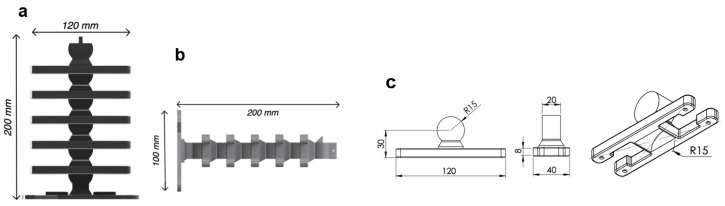
The musculoskeletal system consisted of six rigid segments: (**a**) front and (**b**) side views show the overall dimensions; and (**c**) shows the dimensions of a rigid segment, which consisted of four ribs. The fibers ran through holes in each segment to transmit the forces in hyperelastic material for actuation. The disk–socket joint enables rotation for undulation. The socket is the bottom cavity in the (**c**) isometric view.

The overall musculoskeletal system’s height and width were 200 mm and 120 mm, respectively (Figure 5a). The dimensions of a rigid segment are provided in Figure 5b. Each rigid segment consisted of a disk–socket joint with two ribs on each side. The system’s total mass was approximately 220 g. All the rigid segments were allowed to rotate except for the bottom segment. The musculoskeletal system was attached to a rigid base metal plate to ensure a fixed boundary condition at the bottom segment.

Fused deposition modeling (FDM) was utilized to print each rigid segment separately using an Ultimaker II 3D printer with an 8 mm nozzle head. The rigid segments were 3D printed with PLA filament, a thermoplastic polymer material. It was necessary to 3D print the PLA material of the ribs along the directions of +X and −X to sustain the normal bending stresses induced by the resistive forces in the soft material [46].

The final assembly of the musculoskeletal system was achieved by submerging the rigid segments with Kevlar fibers in the hyperelastic material and then allowing them to cure. We selected Ecoflex for fabricating the soft material due to its flexible casing properties and low viscosity, which enabled us to obviate most of the air bubbles trapped inside the soft material via agitation before curing. The soft material was cured at room temperature, which eliminated any potential thermal expansion issues.

**Figure 6:**
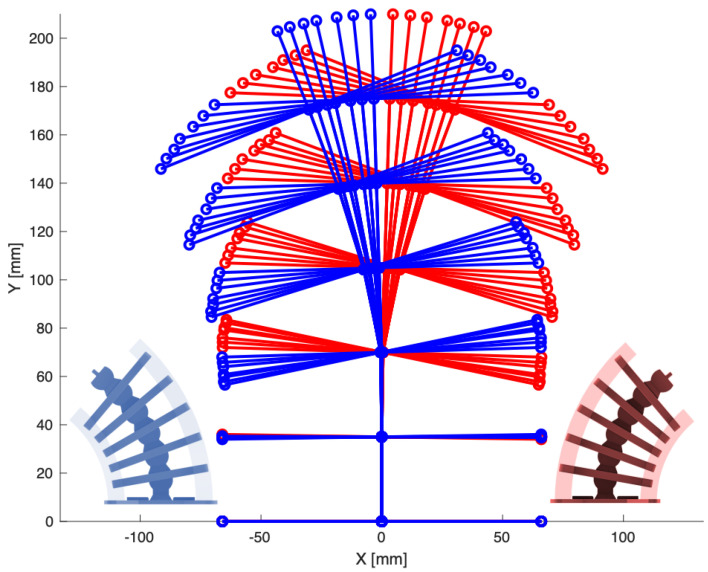
Using imaging analysis, displacements were obtained experimentally for each rigid segment at the center and at the rib endpoints (highlighted in markers). Right (+X) and left (−X) are shown in red and blue, respectively.

The disk–socket joint design of the rigid segments permitted only tangential rotation, as shown in Figure 6. Inserting fibers through the neutral line of the rigid segments ensured that bending occurred only at the pivot points—located at the center of each rigid segment to simplify kinematic analysis [47]. This connection can be thought of as the spinal cord in the vertebrae.

Similar to a snake’s vertebrae, the rotation of each rigid segment induced the collective bending of the complete system, as shown in Figure 6. Therefore, the design strategy facilitated distributed actuation of the system through the soft material by simply applying tension forces carried by the fibers to rotate the rigid segments and cause stretch and contraction in the soft material. The material snapped back to an equilibrium state upon removing the tension forces—releasing the stored energy and returning the spine to the center line.

## 3. Kinematics Development

**Figure 7:**
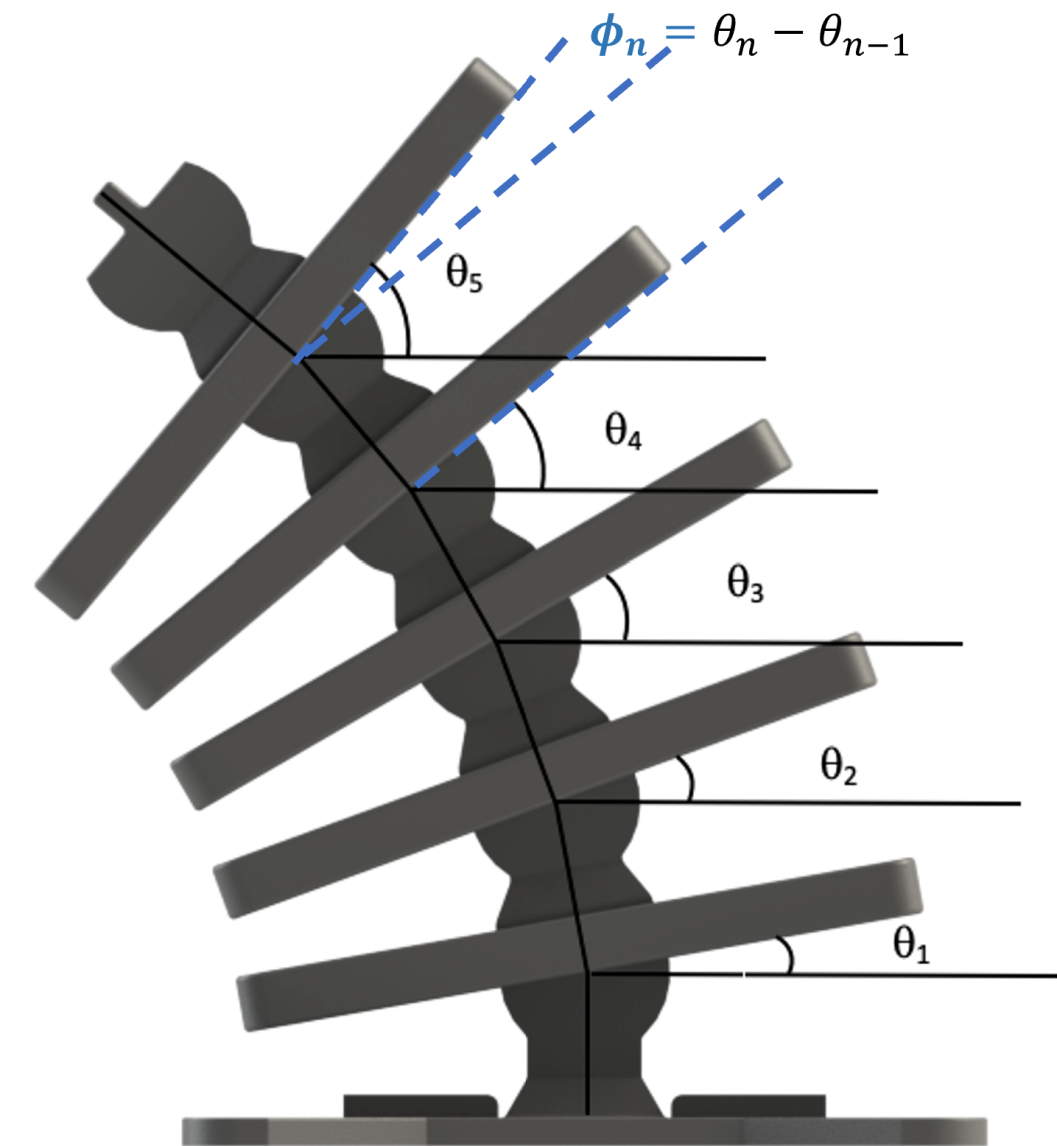
The angles $\theta_{n}$ are the degrees of freedom of the musculoskeletal system with respect to the horizontal axis. The bottom rigid segment is fixed. The angle $\phi_{n}$ is the displacement between segments *n* and n−1.

Development of the kinematics required identifying the minimal degrees of freedom to describe the musculoskeletal system’s full motion. The undulation displacement for each segment *n* is expressed by the angle, $\theta_{n}$, with respect to the horizontal axis, as depicted in Figure 7.

**Figure 8:**
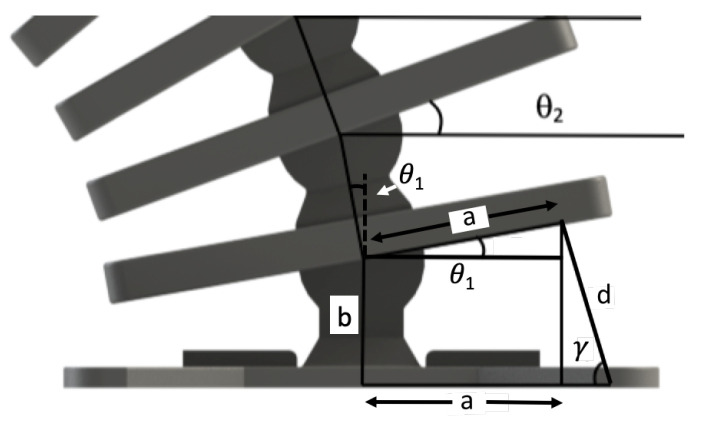
Local geometric parameters used to develop the kinematics.

The angle γ in Figure 8 is of the fiber at the *n*th segment with respect to the vertical axis. The distance between pivot points *n* and n−1 is *b*, which is fixed. The horizontal distance *a* is from a pivot point to the side fibers. The resultant length between ribs *n* and n−1 is *d*. Due to the design symmetry of the system (Figure 8), the tip displacements of the *n*th rib at *a* can be calculated as follows:

(1) $$\left\{\begin{array}{c} x_{rn}\\ y_{rn} \end{array}\right\}=\left\{\begin{array}{c} x_{pn}\\ y_{pn} \end{array}\right\}+a\left\{\begin{array}{c} \cos\theta_{n}\\ \sin\theta_{n} \end{array}\right\}$$

where $x_{pn}$ and $y_{pn}$ are the pivot point displacements of rigid segment *n* rotating in the xy plane. The net angular displacement between segments *n* and n−1 is:

(2) $$\phi_{n}=\theta_{n}-\theta_{n-1}$$

## 4. Experimental Evaluation

**Figure 9:**
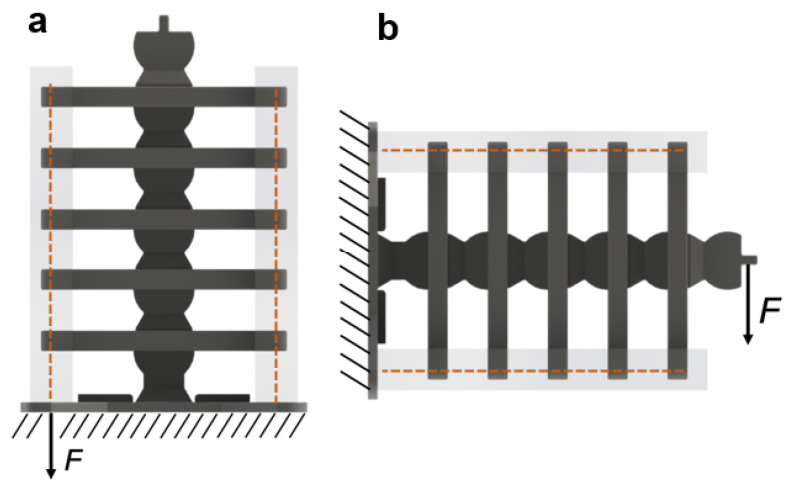
Loading configurations in the (**a**) vertical and (**b**) horizontal directions. F is the applied load. The orange dash lines indicate the internal fibers.

Two sets of experiments were performed to evaluate the energy storage and release of the musculoskeletal system. Each experiment was conducted seven times to ensure repeatability. The first set examined the elastic energy capacity of the actuator by measuring the range of bending motion due to a vertical force applied to the side fibers running through the ribs and a bending force at the maximum moment arm of the actuator, as shown in Figure 9a,b, respectively. In the second set, the musculoskeletal system was exposed to impulse forces, causing it to undulate. The system’s structural damping and the linear and nonlinear structural stiffness were calculated in Section 5.

**Figure 10:**
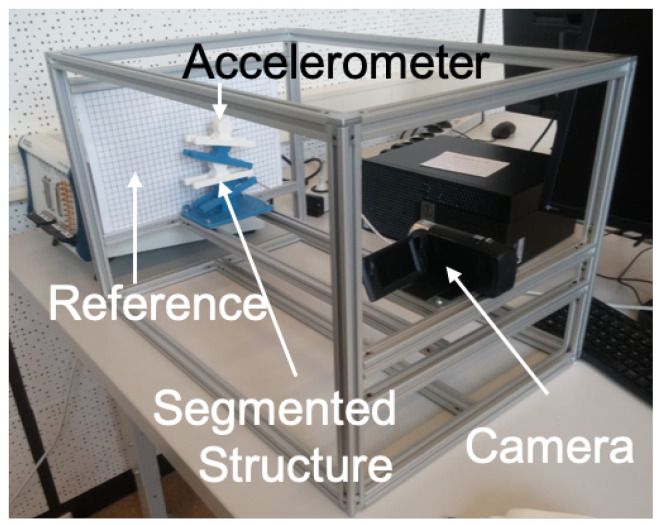
The musculoskeletal system and the video camera were secured to a rigid test frame to maintain accurate measurements. A scale reference was positioned behind the system to facilitate reference for the tracking software.

The metal base of the musculoskeletal system was rigidly fixed to a test frame with four bolts. The test was constructed with 80/20 T-slot aluminum, as shown in Figure 10. The musculoskeletal system bending motion was recorded using a video camera (Model: HDR-CX405, SONY, Tokyo, Japan). The recording was set for a full high-definition option with 60 frames/second. Both the musculoskeletal system and camera were rigidly secured to the test frame to maintain accurate measurements.

### 4.1. Energy Storage Assessment

Video-tracking software called Tracker was utilized to extract the displacement data from videos for each image frame [6]. An example video is provided as a Video S2. Bending displacement was achieved by applying force in the vertical configuration and then switching to the horizontal configuration by rotating the musculoskeletal system by 90°, as illustrated in Figure 9a,b, respectively. The maximum applied force was 29.4 N for both configurations.

**Figure 11:**
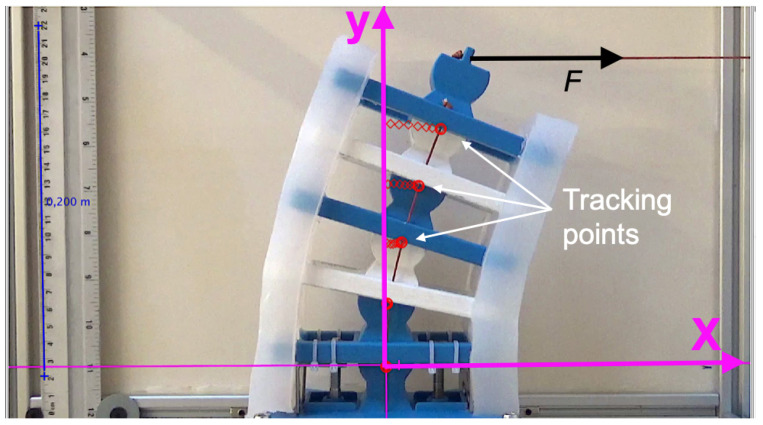
Image tracking of musculoskeletal system motion was obtained using an app called Tracking. The ruler on the left side was utilized for scaling the experimental images.

Using Tracker, the pivot points were identified and collected for each video frame, as shown in Figure 11. The horizontal and vertical axes were specified in each frame with a scale reference. The displacement measurements were converted to data format files to facilitate kinematic analysis using a customized MATLAB—code provided in Code S3 File. The code calculated the change in the angle of every rigid segment relative to the horizontal reference line for every frame, and the net angle, $\phi_{n}$. The change in the distance *d* was obtained from the angle calculations. The change in distance *d* was utilized to calculate the stretch in the soft material, as detailed in Section 5.

### 4.2. Energy Release Assessment

Assessing the energy release required obtaining dynamic parameters due to applying an impulse force, namely structural stiffness and damping. An acceleration sensor was utilized since the video camera lacked the required framerate needed to capture high-frequency undulations. Acceleration measurements were obtained using a lightweight (≈1.0 g) tri-axial sensor (5.0 mV/g sensitivity, Model 3133A3, Dytran Instruments, Inc., Chatsworth, CA, USA). The sensor was placed at the center of the top rigid segment of the system, as illustrated in Figure 10. The impulse force was delivered to the top segment in the horizontal direction (X) using a miniature modal hammer (21.1 mV/N sensitivity, Model 2302-100, Meggitt Endevco, Irvine, CA, USA). The sensor and impulse force placement at the top segment provided the most accurate measurements because the motion was maximum. The response was normalized by the input force.

## 5. Mechanics of Elastic Energy Storage–Release

### 5.1. Elastic Storage in Hyperelastic Material

It is assumed that silicone material behaves in an isotropic manner [36]. Based on the uniaxial nonlinear elastic deformation range, the stretch ratio in the axial direction can be calculated as follows:

(3) $$\lambda_{1}=\frac{l}{l_{o}}$$

The engineering strain is ε=λ−1. The principal stretch is [48]:

(4) $$J=\lambda^{2}+\frac{2}{\lambda }$$

**Figure 12:**
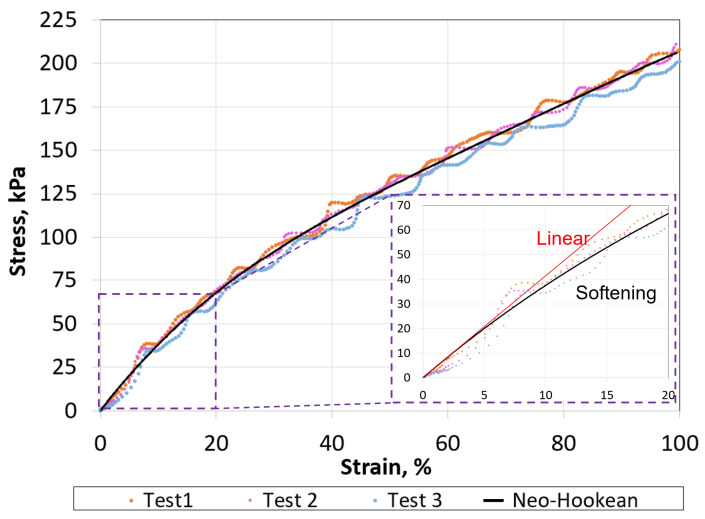
Neo-Hookean hyperelastic constitutive model shows good agreement with the experimental stress–strain response of the soft material. The inset plot shows the nonlinear response at low strains, which is the softening (black curve) region in Figure 4. The straight red line highlights the nonlinear response.

The silicone stress–strain response was obtained from uniaxial tension testing for three samples, as shown in Figure 12. Each sample was stretched up to 100% strain. The neo-Hookean model was applied to capture the soft-material deformation with reasonable accuracy (Figure 12). The neo-Hookean model is based on a strain-energy-density function given by the following expression [6]:

(5) $$S=\frac{E_{o}}{6}\left(J-3\right)$$

From Figure 12, the elastic constant at the initial stretch, $E_{o}$, was 3.602 MPa. Substituting *J* into *S* and differentiating with respect to λ, the uniaxial nominal stress becomes:

(6) $$\sigma =\frac{E_{o}}{3}\left(\lambda -\lambda^{-2}\right)$$

Knowing $\sigma =E_{e}\varepsilon$, the variable elastic modulus as a function of the stretch can be calculated as follows:

(7) $$E_{v}=\frac{E_{o}}{3}\left(\frac{\lambda -\lambda^{-2}}{\lambda -1}\right)$$

Each segment was approximated as a nonlinear rotational spring element, which was a concentrated variable-bending stiffness. Each rib displacement due to bending can be approximated as $\Delta x_{n}=a\sin\phi_{n}$, since $\phi_{n}$ is relatively small compared to the total displacement of the system. Therefore,

(8) $$\lambda =\frac{l_{o}+a\phi_{n}}{l_{o}}$$

**Figure 13:**
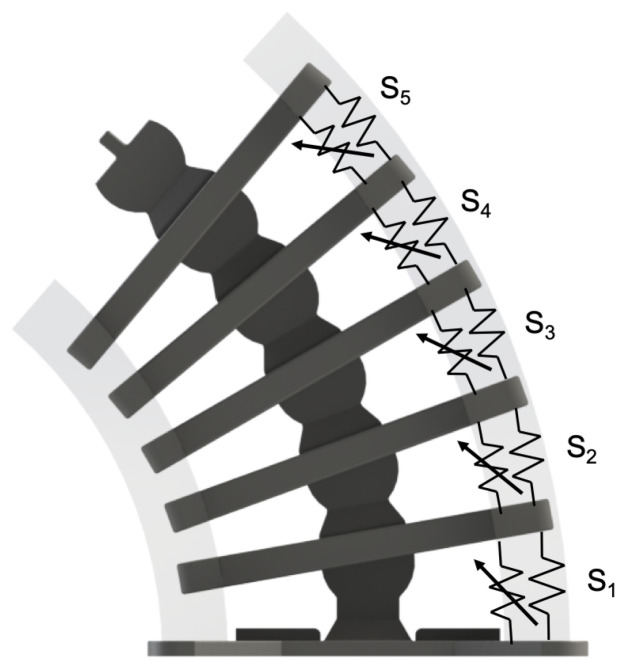
The soft material model was expressed as linear and nonlinear springs ($S_{n}$) using constant and tunable resistive elements, respectively.

The overall bending displacement exhibited linear and nonlinear quadratic deformations. Therefore, the restoring force can be expressed as the combined effect of linear and nonlinear springs:

(9) $$F_{n}=\left(k_{n,1}+k_{n,2}\phi_{n}\right)\phi_{n}=S_{n}\phi_{n}$$

where $S_{n}$ is the equivalent nonlinear stiffness for the *n*th spring, as shown in Figure 13—the linear and nonlinear stiffness values are $k_{n,1}$ (spring without arrow) and $k_{n,2}$ (spring with arrow), respectively. Equation (9) was utilized as an analytical model to calculate the force–displacement for each segment *n*. The stored elastic energy $U_{n}$ for the *n*th spring can be obtained by integrating the force in (9) with respect to the displacement [49]:

(10) $$U_{n}=\frac{1}{2}k_{n,1}\phi_{n}^{2}+\frac{1}{3}k_{n,2}\phi_{n}^{3}=(\frac{1}{2}k_{n,1}+\frac{1}{3}k_{n,2}\phi_{n})\phi_{n}^{2}$$

The total elastic energy stored in the system can be computed by summing all $U_{n}$.

(11) $$U_{total}=\sum_{n}U_{n}$$

### 5.2. Energy Release in Undulation

The entire system resonance frequency ($f_{r}$) and damping ratio (η) were obtained experimentally from the acceleration measurements after each impulse event from the frequency domain data. The resonance frequency can be thought of as the rate at which the musculoskeletal system will oscillate at its dominating mode shapes [50]. Bending was the first dominating mode shape, i.e., the mode with the lowest $f_{r}$. The undulation frequency is given by [51]:

(12) $$f_{r}=\frac{1}{2\pi }\sqrt{\frac{k_{e}}{m_{e}}}$$

In the context of structural dynamics, the effective stiffness and mass for the first mode of the entire musculoskeletal system are $k_{e}$ and $m_{e}$, respectively [52]. For clarity, the effective (or moving) mass is intrinsic to a mode shape and is not the same as the total mass of the system, since each part in the structure will move differently. Similarly, $k_{e}$ is the effective stiffness of all components forming specific shapes for different harmonics. Additionally, η is the ratio of the energy dissipated, $E_{d}$, to the maximum elastic energy stored. Therefore, the total energy released for the entire system can be calculated as follows:

(13) $$E_{r}=U_{total}-E_{d}=U_{total}\left(1-\eta \right)$$

The damping ratio can be estimated at each resonance frequency from the amplification factor as follows [53]:

(14) $$\eta =\frac{1}{2Q_{r}}$$

The amplification factor $Q_{r}$ is computed using the half-power bandwidth method [53]:

(15) $$Q_{r}=\frac{f_{r}}{\Delta f}$$

where Δf is the frequency bandwidth between −3 dB points on the frequency response function, also known as the half-power points. For additional interest in the method, the reader is encouraged to reference [53].

## 6. Results and Discussion

### 6.1. Elastic Energy Storage

**Figure 14:**
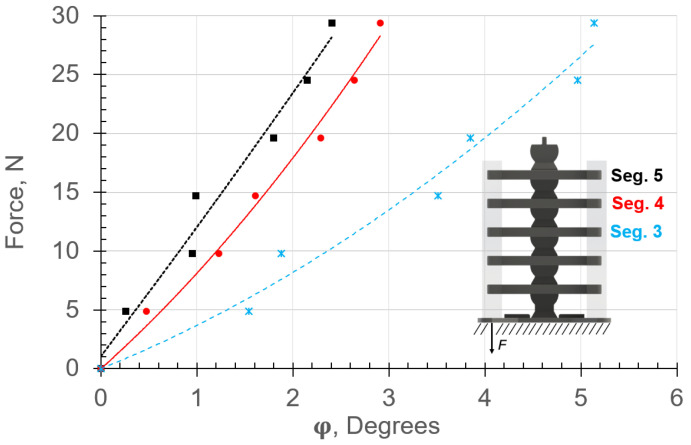
Force–response results for vertical loading obtained by applying force at the end of the left-side fibers. Experimental and analytical (from (9)) results are shown in markers and lines, respectively. Note the nonlinear bending behavior due to contraction of the soft material on one side and stretch on the other.

**Figure 15:**
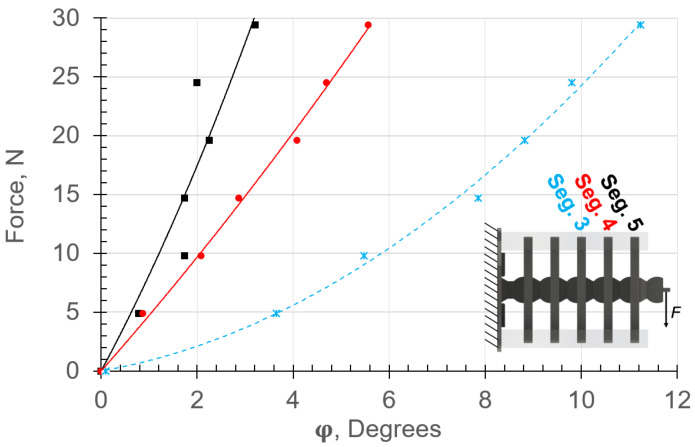
Force–response curves for horizontal loading obtained by applying force at the end of the top rigid segment. Experimental and analytical (from (9)) results are shown in markers and lines, respectively. Note the nonlinear bending behavior due to contraction of the soft material on one side and stretch on the other.

The experimental and analytical range of motion, $\phi_{n}$, due to the application of forces in the vertical and horizontal directions are provided in Figure 14 and Figure 15, respectively. The experimental and analytical results are shown in markers and lines, respectively. The angle $\phi_{n}$ and force *F* are expressed in degrees and N, respectively. The displacements were calculated using (9), where the force–displacement relationships included the material nonlinearity.

Inducing vertical and horizontal loads caused compression and tension stresses in the left and right soft muscles (Figure 6), respectively. It can be observed that the analytical results are in good agreement with the experimental data for both vertical and horizontal configurations (Figure 14 and Figure 15, respectively). The displacement $\phi_{n}$ in both cases (Figure 14 and Figure 15) differed for each rigid segment and was nonlinear except for Top Segment 5, which behaved linearly due to lower stretch/contraction in the soft materials. This expected behavior resembled nature because each soft muscle, $S_{n}$, between two rigid segments deformed differently. Hence, in biological musculoskeletal systems, the muscles tend to be thicker and highly stretchable in areas with high bending [9].

The nonlinear deflection behaviors were attributed to the hyperelastic material’s capability of aggregating significant elastic energy as a function of the large geometric displacement in the system. Notice the change in the resistance force in the spring segments $S_{3}$ and $S_{5}$ as a function of $\phi_{n}$. The highest nonlinear displacement occurred in the lowest soft element. The lower elements carried more bending loads than the top ones. Therefore, the maximum energy storage capacity occurred in the soft muscles (springs) closer to the base due to the high stretch on one side and compression on the other. Additionally, $S_{3}$ experienced dynamic hardening as the tension force was increased, while $S_{4}$ and $S_{5}$ experienced dynamic softening. The stretch in $S_{3}$ due to the horizontal loading configuration was higher than that for the vertical configuration. Since the moment arm in the horizontal loading configuration was higher than that in the vertical case, the stresses were higher as well.

**Figure 16:**
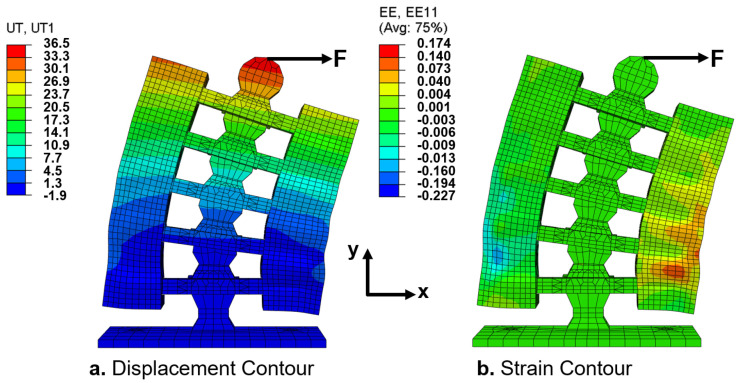
Contours of the displacement in the *X*-direction (**a**) and of the principle strain, $\varepsilon_{xx}$, due to a 29.4 N force applied to the end of the top rigid segment; (**a**) the maximum displacement (in mm) is $\phi =11.0^{{}^{\circ}}$, which is 4.4% less than the maximum experimental angle of 11.5°; (**b**) the strain is highest in the hyperelastic materials between the rigid segments (ribs), where energy is stored.

Finite element analysis (FEA, using Abaqus CAE-SIMULA, Dassault Systemes, Vélizy-Villacoublay, France) was performed to gain additional insights into the elastic energy storage mechanics, as shown in Figure 16. The analysis was conducted for the horizontal loading configuration up to 29.4 N. We assumed that the rigid and soft materials were elastic, isotropic, and incompressible. However, the hyperelastic material constitutive model supplied to ABAQUS was based on the experimental stress–strain data in Figure 12. The effect of geometric nonlinearity was included in the model. Quadratic brick 20-node (C3D20) elements were utilized to mesh the rigid segments. For the hyperelastic material, 20-node quadratic brick hybrid (C3D20H) elements were chosen to remedy volume–strain locking due to the silicone’s high Poisson ratio of 0.48 [48]. Future FEA studies will be required to understand the effect of hysteresis on energy storage–release in the system over time.

The contours of the transverse displacement in the *X*-direction and principle strain, $\varepsilon_{xx}$, are reported in Figure 16 for the 29.4 N force (Figure 15). The maximum deflections obtained from the simulations were in good agreement with the experimental results. The largest error was ∼4.4% at 29.4 N, where the FEA and experimental deflections were 11.0° and 11.5°, respectively (Figure 16). The deviation between the simulations and experiments could be attributed to the low geometric tolerances of 3D printing and silicone casting. Figure 16 shows that the tension and compression in the hyperelastic material were maximum and minimum between the lower and top ribs, respectively. For example, the strains in the stretched and compressed regions of the $S_{2}$ spring were approximately 17.4% and 22.7%, respectively. On the −X side, the elastic stretch caused distributed strain in the nonlinear material from 2% to a maximum 22.7%, where substantial elastic necking occurred. Conversely, the strain in the compressive side was more distributive than that in the stretched side. This is common behavior in hyperelastic materials [48]. However, including compressive tests of the hyperelastic material may remedy the deviation between the FEA and experimental results.

**Figure 17:**
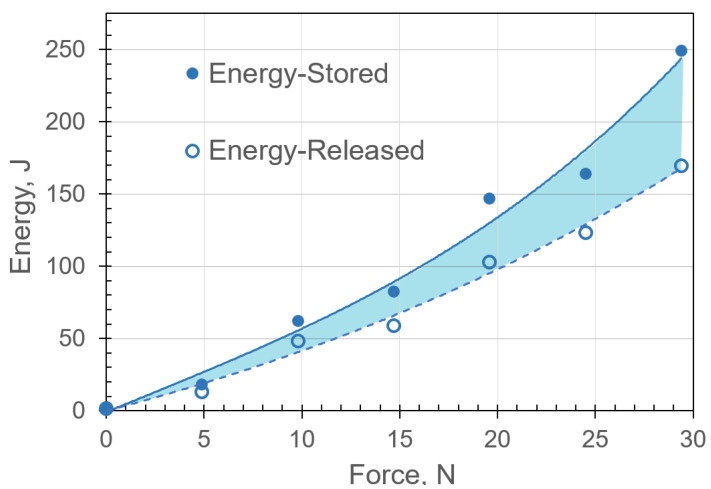
Total elastic energy storage for the musculoskeletal system in the bending load configuration. The energy released was the energy stored minus the energy dissipated. Experimental and analytical (from (11)) results are shown in markers and lines, respectively.

Estimating elastic energy storage was achieved by calculating the forces required to restore the assembly to its equilibrium state, i.e., to the assembly’s centerline. The total elastic energies stored and released were obtained using Equations (10) and (11) and included the contribution of the linear and nonlinear stiffness. The total stored energy from the experimental and analytical results as a function of the applied force were in good agreement, as shown in Figure 17. It can be observed that the stored elastic energy increased considerably in the nonlinear region as the applied force was increased. Hence, increasing the force increased the stretch–contraction in the hyperelastic material, and energy aggregation increased nonlinearly. Energy efficiency was approximately 68% for the maximum displacement of the entire system in the vertical bending configuration. Efficiency was calculated as the ratio of the energy released and injected. The energy dissipation can be attributed to the hyperelastic material’s damping. Detailed discussion is provided in Section 6.2.

### 6.2. Elastic Energy Release

**Figure 18:**
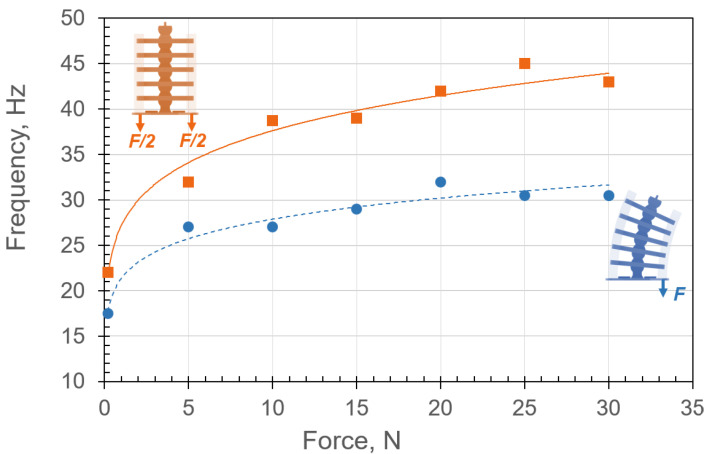
Undulation frequency for single- and double-sided tension configurations in blue and orange, respectively. The frequency appears to plateau asymptotically as the internal force is increased for both systems.

The resonance frequencies, $f_{r}$, due to the musculoskeletal system’s free oscillation (undulation) motion at different applied loads were calculated and are shown in Figure 18. After inducing an impulse force, the system was free to undulate about the equilibrium position at its resonance frequency, which is the number of oscillations per second. Therefore, the resonance frequency was an ideal measure of the speed of undulation.

Free-oscillation-response data obtained from the modal impact experiments were recorded in the time domain and then transformed into the frequency domain [54,55]. The experiments were conducted for up to 200 Hz frequency with 0.5 Hz resolution. The natural frequency was estimated for the musculoskeletal system in two configurations: single- and double-sided tension of the soft materials, as shown in Figure 18. Applying tension force to the soft material caused contraction, which increased the resonance frequency (or equivalent stiffness).

It can be concluded that increasing the tension in the muscles increases the undulation speed. For example, the undulation speed in the double-sided tension case was approximately 45 Hz due to applying a 29.4 N force, which meant that the system undulated with 2×43=86 center-crossings per second. In the absence of internal forces in the soft material, the undulation was 2×22=44 center-crossings per second. Note an initial increase in the force increased the undulation frequency significantly. However, the undulation frequency tended to plateau asymptotically as the internal force surged. This important finding indicates that the system is indeed nonlinear hybrid compliant, where the plateau is the softening–hardening transition in the stiffness (Figure 4). It is expected that a further increase in the applied force will cause the system to harden, and the force–displacement will become cubic (Figure 4). This behavior is consistent with the periodic motion seen in animals, where muscle stiffness varies to facilitate actuation over a broad natural frequency envelope [9]. The reason for actuating near or at the natural frequency is to maximize the response amplitude [56].

**Figure 19:**
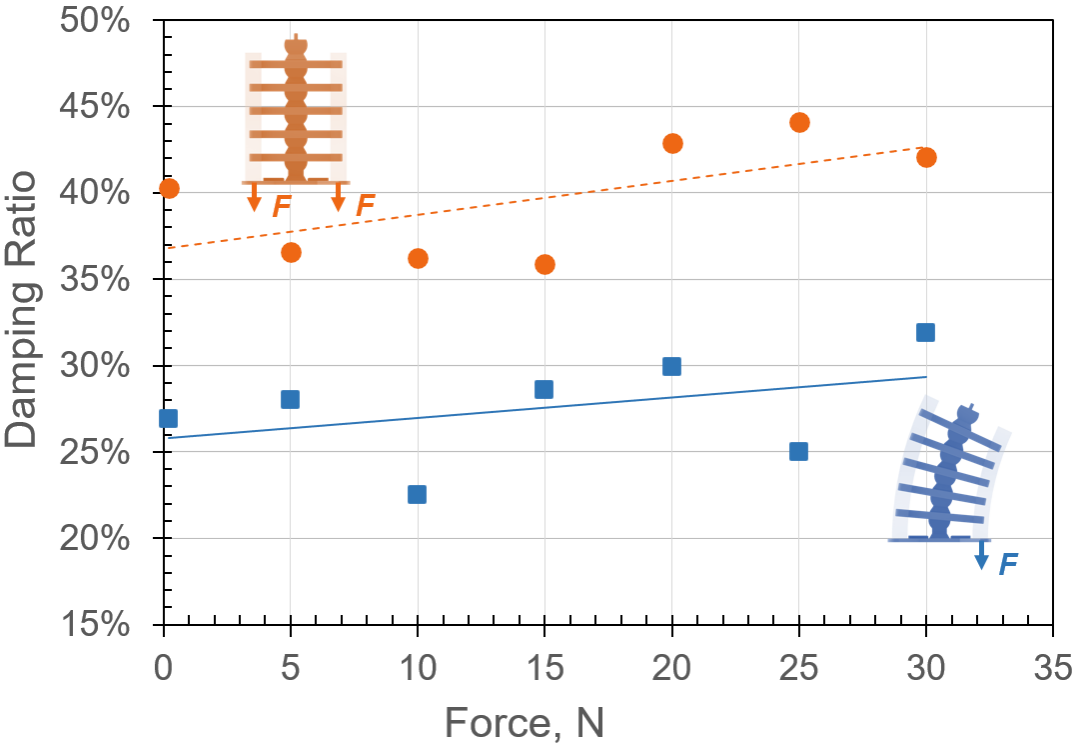
Damped undulation due to energy dissipation for single- and double-sided tension configurations in blue and orange, respectively. The damping ratio appears to increase linearly as the internal force is increased for both systems.

The corresponding damping due to applied forces in single-sided and double-sided tension configurations are reported in Figure 19. Both configurations show that an increase in the internal forces increased the overall structural damping. As expected, the system was highly damped due to energy absorption by the hyperelastic materials [6] and frictional forces between the joints [28]. For an applied internal force of 29.4 N in the single- and double-sided tension configurations, the damping ratios were approximately 32% and 42%, respectively. It is important to point out that the stiffness appeared to plateau asymptotically as the applied force was increased. While damping in Figure 19 appears to increase linearly, it should plateau as we increase the load, since it cannot continue this trend forever. The linear appearance could be due to the scatter in the data, which is common when estimating the damping of vibratory systems [37]. This behavior is also consistent with the soft material stress–strain response (Figure 12).

The damping ratios were utilized to estimate the energy dissipation (Figure 17). It can be observed that the release speed nearly doubled as the energy stored in the hyperelastic materials reached 250 J. This is consistent with the explosive motion of vertebral undulating animals.

Future research will include investigating the effects of geometric and material nonlinearity in various dynamic modes including steady-state motion. The goal is to understand the competing mechanisms between these nonlinearities, in which material nonlinearity tends to cause dynamic softening or compliance, and geometric nonlinearity promotes dynamic hardening [37]. The contribution of friction is another important topic that will be addressed in future studies. More precisely, design principles are needed to describe the variation in friction due to tuning the structural stiffness with actuation forces.

## 7. Conclusions

In this study, the mechanics of energy storage–release were demonstrated for a nature-inspired musculoskeletal system that consisted of 3D-printed rigid segments (bones) connected with soft hyperelastic material (muscles). The energy efficiency of the musculoskeletal system was approximately 68% to bend the structure from a vertical configuration to a 158 mm radius-of-curvature. Efficiency was defined as the ratio between the released and stored energy. energy losses were attributed to the soft material’s high damping and to joint friction between the rigid segments in the musculoskeletal system. While contraction in the soft material increased by approximately 6%, an increase of more than 100% in the structural stiffness counteracted the negative effect of damping.

Nature-inspired musculoskeletal systems hold great promise as an alternative to current soft actuators that struggle to go beyond 30% energy efficiency. Combining rigid segments and soft materials in designing a musculoskeletal system was key to achieving these advantageous features. Utilizing 3D printing allowed us to easily apply geometric segmentation and nonlinear materials, as seen in nature, to demonstrate combined high elastic energy storage and range of motion in the system while maintaining structural stability.

## Data Availability

The data presented in this study are available on request from the corresponding author. The data are not publicly available due to the large size of the files.

## Supplementary Materials

The following supporting information can be downloaded at: https://www.mdpi.com/article/10.3390/mi13101783/s1. Video S1: Motion of the Musculoskeletal System, Video S2: Tracking the Deflection Motion, Code S3: Kinematics of the Musculoskeletal System.
