# Supplementary figures and images for: Hybrid Compliant Musculoskeletal System for Fast Actuation in Robots
Source: Micromachines (Basel). 2022 Oct 20;13(10):1783. doi: 10.3390/mi13101783 (PMC9611504; doi:10.3390/mi13101783)

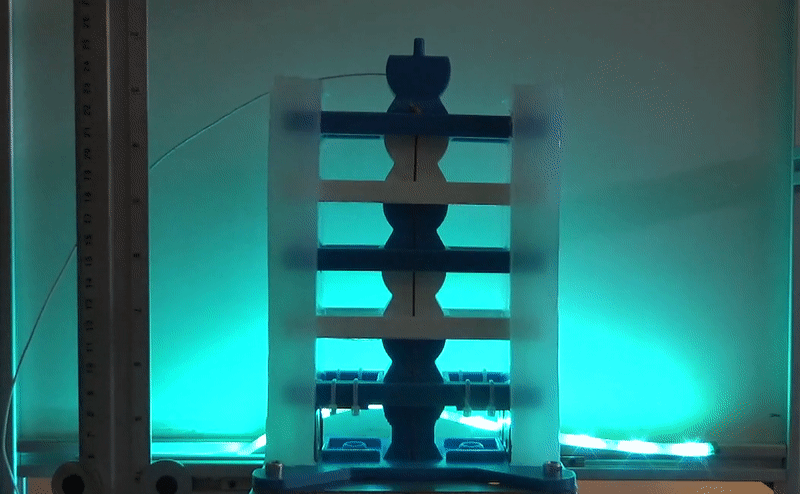

Supplement: Supplementary file 1 [file micromachines-13-01783-s001.zip › Video S1.gif]

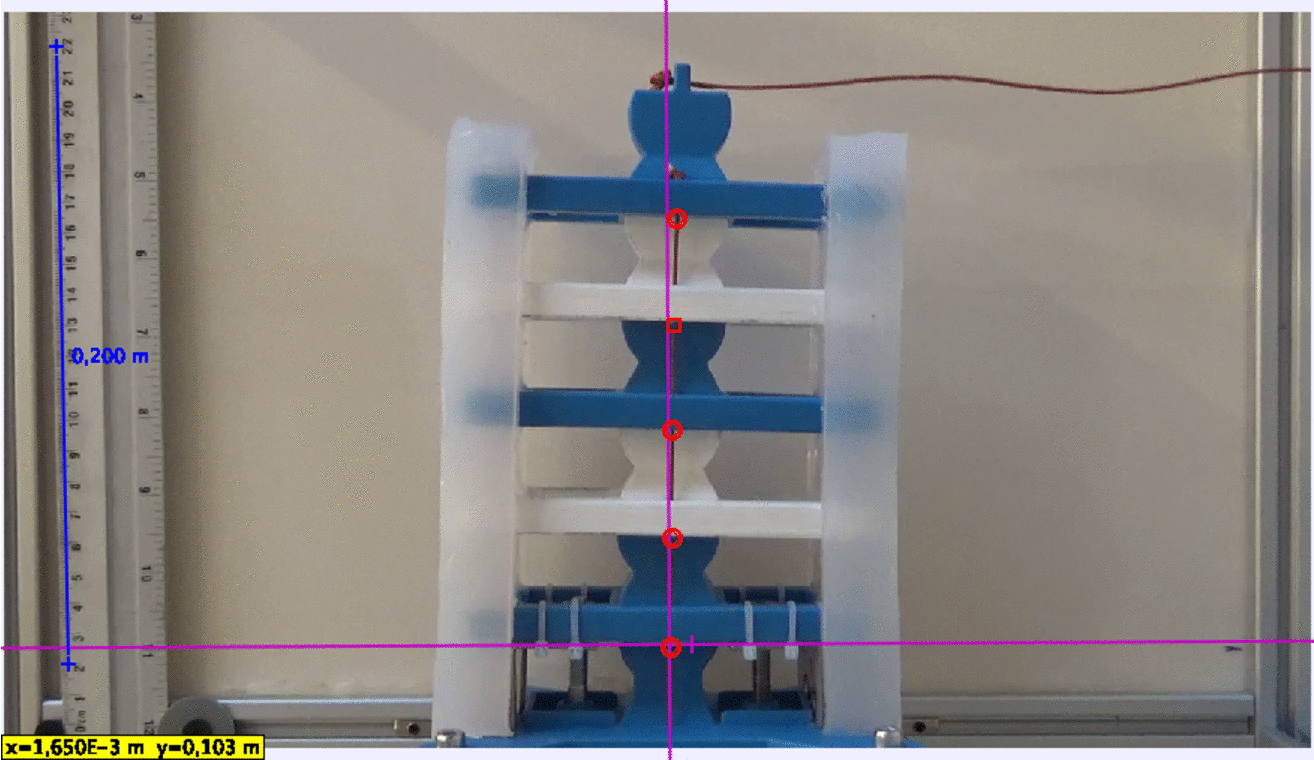

Supplement: Supplementary file 1 [file micromachines-13-01783-s001.zip › Video S2.gif]
